# Deep Learning-Based Automatic Muscle Segmentation of the Thigh Using Lower Extremity CT Images

**DOI:** 10.3390/diagnostics15222823

**Published:** 2025-11-07

**Authors:** Young Jae Kim, Ji-Eun Kim, Yeonho Park, Jae Won Chai, Kwang Gi Kim, Ja-Young Choi

**Affiliations:** 1 Gachon Biomedical & Convergence Institute, Gachon University Gil Medical Center, Incheon 21565, Republic of Korea; kimyj@gilhospital.com; 2 Department of Radiology, Yeouido St. Mary’s Hospital, Seoul 07345, Republic of Korea; je0113kim@gmail.com; 3 Department of Radiology, Seoul National University Hospital, Seoul National University College of Medicine, Seoul 03080, Republic of Korea; parkyeonhi@gmail.com; 4 Department of Radiology, SMC-SNU Boramae Hospital, Seoul 07061, Republic of Korea; chaijw@gmail.com; 5 Department of Biomedical Engineering, College of Medicine, Gachon University, Incheon 21999, Republic of Korea

**Keywords:** thigh muscle, deep learning, computed tomography, segmentation, volumetry

## Abstract

**Background/Objectives:** Sarcopenia and muscle composition have emerged as significant indicators in the fields of musculoskeletal and metabolic research. The objective of this study was to develop and validate a fully automated, deep learning-based method for segmenting thigh muscles into three functional groups (extensor, flexor, and adductor) using non-contrast computed tomography (CT) images and to quantitatively evaluate the thigh muscles. **Methods:** In order to ascertain the most efficacious architecture for automated thigh muscle segmentation, three deep learning models (Dense U-Net, MANet, and SegFormer) were implemented and subsequently compared. Each model was trained using 136 manually labeled non-contrast thigh CT scans and externally validated with 40 scans from another institution. The performance of the segmentation was evaluated using the Dice similarity coefficient (DSC), sensitivity, specificity, and accuracy. Quantitative indices, including total muscle volume, lean muscle volume, and intra-/intermuscular fat volumes, were automatically calculated and compared with manual measurements. **Results:** All three models exhibited high segmentation accuracy, with the mean DSC exceeding 96%. The MANet model demonstrated optimal performance in internal validation, while the SegFormer model exhibited superior volumetric agreement in external validation, as indicated by an intraclass correlation coefficient (ICC) of at least 0.995 and a *p*-value less than 0.01. **Conclusions:** A CT-based deep learning framework enables accurate and reproducible segmentation of functional thigh muscle groups. A comparative evaluation of convolutional attention- and transformer-based architectures supports the feasibility of CT-based quantitative muscle assessment for sarcopenia and musculoskeletal research.

## 1. Introduction

Sarcopenia, defined as a decrease in body muscle mass, is a salient health problem in an aging society. In recent years, the clinical significance of sarcopenia in patients with various conditions has garnered increased recognition. In the field of oncology, the assessment of sarcopenia has been demonstrated to offer substantial prognostic insights for patients [1,2]. Furthermore, numerous studies have been published recently examining the association between osteoarthritis and sarcopenia. The condition of the thigh muscles around the knee (particularly the quadriceps muscle) has been demonstrated to be associated with the severity of symptoms and the progression rate of knee osteoarthritis [3,4,5]. As has been previously established, the loss of muscle mass is indicative of a decline in muscle size and strength, as well as significant functional consequences [6]. A recent study indicated a strong correlation between postoperative outcomes of total knee arthroplasty and preoperative functional levels [7,8].

Consequently, it is imperative to possess the capability to precisely quantify muscle volume and to discern alterations in muscle quality, including atrophy and intramuscular fat infiltration. To date, dual energy X-ray absorptiometry (DXA) has been the primary tool used to assess body composition [9]. Cross-sectional areas at the abdominal and thoracic computed tomography (CT) scan have been shown to be linearly related to the whole-body muscle mass [10]. While DXA is a valuable tool for assessing overall body composition, it has limitations in differentiating between specific fat depots and muscle components. The instrument is incapable of distinguishing between subcutaneous and visceral fat in the trunk or between extra-muscular and intra-muscular fat in the trunk and extremities [11]. Furthermore, DXA is not appropriate for evaluating the function of specific muscles or muscle groups. Its sensitivity to minor changes is limited, rendering it less useful for evaluating short-term post-treatment effects. Additionally, DXA is not equipped to provide information regarding muscle quality or functional capacity.

Magnetic resonance imaging (MRI) has become a widely utilized imaging modality for assessing skeletal muscle mass and composition, owing to its superior soft tissue contrast and its lack of ionizing radiation. A multitude of population-based studies have demonstrated a robust correlation between MRI and metabolic health, as well as between bioelectrical impedance analysis (BIA) and muscle composition. This correlation serves to substantiate MRI as a validated reference method for muscle composition assessment [12,13]. Nevertheless, MRI is comparatively expensive and time-consuming, which restricts its viability for large-scale or routine clinical applications.

CT-based measurement of muscle mass has been demonstrated to be more accurate than that obtained by DXA; however, it is difficult to apply this technique to whole-body muscle mass evaluation. Furthermore, the method by which the cut-off range of muscle attenuation is established has the potential to influence variability in muscle mass measurements, as the value obtained from a single section may not be representative of the subject’s overall muscle mass. Recently, a standardized threshold range of muscular attenuation (from −30 to +150 Hounsfield units (HU)) has been presented as a means to facilitate volume measurement [14]. A comparison of MRI and CT reveals several notable advantages. CT is more widely available, less expensive, and already integrated into many clinical workflows. When combined with automated segmentation, CT facilitates a reproducible and quantitative assessment of muscle and fat composition. Consequently, a CT-based deep learning approach has the potential to offer a practical and scalable alternative to MRI for muscle-composition analysis.

The application of deep learning in the domain of radiology has been a subject of considerable research, particularly in the areas of lesion classification, detection, and segmentation [15,16]. The application of deep learning has been demonstrated to yield superior performance in the domain of computer vision, thereby surpassing the existing limitations imposed by conventional machine learning algorithms [17]. In 2019, Haquer et al. undertook a study to segment 10 distinct thigh muscles using a 2.5D U-Net architecture on CT scans. The dataset comprised a total of 48 CT scans, of which 38 were utilized for training purposes, while the remaining 10 were allocated for testing. The findings indicated an average dice similarity coefficient (DSC) of 91.18% [18]. In 2022, Agostini and colleagues initiated a project to segment 12 distinct types of thigh muscles using a deep neural network (DNN) architecture, a technique within the realm of deep learning, applied to MRI scans. The DNN model was trained on 110 MRI scans from 44 subjects and subsequently tested on 10 MRI scans from a separate set of 10 subjects. The findings revealed an average DSC of 87.89% [19]. In 2023, Gaj et al. [20] conducted a study to segment three types of thigh muscles in MRI using the U-DenseNet architecture. The model was trained on a total of 16 MRI scans, and four-fold cross-validation was performed. The results demonstrated an average DSC of 97% [20]. As demonstrated by the aforementioned studies, there have been numerous attempts to segment thigh muscles using deep learning techniques. However, the majority of these studies have relied on MRI. Although MRI provides superior contrast for soft tissues compared to CT, facilitating muscle segmentation, it has the disadvantage of a longer acquisition time compared to CT and necessitates a meticulously designed protocol for each study. A number of studies have sought to segment thigh muscles using CT imaging. However, the efficacy of this approach has been observed to be somewhat inferior to that of conventional MRI methods.

This discrepancy can be attributed primarily to the lower soft tissue contrast and thinner slice thickness of CT images. These characteristics render intermuscular boundaries more ambiguous and manual labeling more challenging, thereby affecting the accuracy of both training and evaluation. Nevertheless, CT is far more accessible in routine clinical settings, faster to acquire, and widely obtained for other indications, allowing opportunistic analysis without additional imaging or radiation. These characteristics render CT a pragmatic and expandable modality for large-scale muscle-composition evaluation, especially in institutions where MRI is not a standard procedure. Consequently, the objective was to develop and validate a fully automated deep learning-based segmentation method capable of achieving reliable performance on non-contrast CT despite these intrinsic modality limitations.

The objective of this study was to implement a deep learning-based technique for automated muscle segmentation and quantitative evaluation, encompassing intramuscular, intermuscular, and subcutaneous fat, as well as total and individual muscle volumes. An additional objective of this study was to develop and validate a fully automated, deep learning-based method for quantitative assessment of thigh muscles using non-contrast CT images.

## 2. Materials and Methods

### 2.1. Data with Training and Internal/External Validation

During the period between January 2017 and December 2019, a total of 136 patients over the age of 19 who underwent non-contrast lower extremity CT scans in our institution were included in the study. The study population included 136 patients, of whom 44 were male and 92 were female, with a mean age of 51.56 years. However, BMI data were not available for this retrospective cohort because the study’s primary focus was on technical development and validation of the segmentation model rather than clinical correlation analysis. The patients included in the study underwent CT for a variety of musculoskeletal indications and exhibited a typical range of body habitus. Patients whose entire femoral muscles (including hip and knee joints) and entire subcutaneous fat are included in the axial scan range on CT images. The present study was approved by the institutional review board of Soul National University Hospital, Republic of Korea (IRB number H-1904-163-1031). It was determined that, given the minimal risk to the patients involved, a waiver of consent was appropriate for this study. The present study was conducted in accordance with the principles outlined in the Declaration of Helsinki. All methods were performed in accordance with the relevant guidelines and regulations.

A total of four patients were excluded from the study due to the following criteria: a documented history of surgical intervention at the femur, hip, or knee joint; and the presence of severe bone deformity in both hips, femurs, and knee joints, attributable to congenital or traumatic causes. Subsequently, all consecutive lower-extremity CT examinations performed for preoperative knee evaluation between January and December of 2018 at a designated external hospital were reviewed. The external validation cohort comprised 40 patients (2 men and 38 women with a mean age of 70.65 years) who met the inclusion criteria. Consequently, 172 patients were included in the present study.

The study exclusively incorporated non-contrast CT scans obtained under standard diagnostic protocols, while low-dose CT images were excluded to ensure the consistency of image quality across datasets. The CT Protocol subsection provides detailed acquisition parameters.

The clinical dataset comprised 46,380 images, including 42,274 images from the institutional database and 4106 images from external hospitals. This dataset encompassed 172 anonymized non-contrast thigh CT scans, extending from the ischial tuberosity to the patellar base. In each of the three groups, a total of 33,692 and 8582 images from our center were assigned to the training set and the internal validation or test set, respectively. The images from the external hospital were used for external validation.

### 2.2. CT Protocol

All CT examinations for the internal dataset were performed with a dual-layer spectral detector CT system (IQon Spectral CT, Philips Healthcare, Best, The Netherlands) at our institution. CT images were obtained using the following acquisition parameters: tube voltages of 120 kVp, a reference tube current-time product of 140~150 mAs effective, an effective rotation time of 0.4 s, collimation of 64 × 0.625 mm, a pitch of 0.391, a field of view (FOV) of 320~400, a reconstruction slice thickness of 2.0 mm, and no gap.

The external validation dataset was retrospectively collected from a designated outside hospital using a multidetector CT system (Ingenuity Elite, Philips Healthcare, Best, The Netherlands). CT images were obtained using the following acquisition parameters: tube voltages of 120 kVp, a reference tube current-time product of 200~360 mAs effective, an effective rotation time of 0.4~0.5 s, collimation of 64 × 0.625 mm, a pitch of 0.9~1.1, a field of view (FOV) of 370~500 mm, a reconstruction slice thickness of 5.0 mm, and no gap.

Despite the fact that these parameters did not align precisely with those employed for the internal dataset, all scans exhibited diagnostic image quality adequate for evaluating thigh muscles and for assessing model generalizability.

### 2.3. Data Manipulation

The digital imaging and communications in medicine (DICOM) file constitutes de-identified personal health information, which is stored using an anonymization program developed in-house. It is imperative that essential information for research, such as study date, be stored by entering a pseudo-key in DICOM. The original patient information and the document stored with the pseudo-key must be stored in a separate encrypted file so that only the responsible researcher can access them. The development of deep learning programs is conducted using an image file that does not contain patient information.

### 2.4. Development Environment

The present study utilized an IBM Power System AC922 (IBM, Armonk, NY, USA) server with four NVIDIA Tesla V100 with NVLink GPUs with 16 GB (NVIDIA, Santa Clara, CA, USA) for the purpose of conducting deep learning. The deep learning process was executed using Python 3.6.9 and Keras 2.3.0 with TensorFlow 1.14.0 serving as the backend, thereby ensuring comprehensive compatibility between the two frameworks. The application of batch normalization layers following each convolutional block was implemented with the objectives of stabilizing the training process and accelerating convergence speed. The operating system in use was Ubuntu 18.04.3. The software was developed using Microsoft Visual Studio (version 2010, Microsoft Corp., Redmond, WA, USA), ITK (version 4.34.1; Insight Segmentation and Registration Toolkit; Kitware Inc., Clifton Park, NY, USA), and VTK (version 5.10.0; Visualization Toolkit; Kitware Inc., Clifton Park, NY, USA).

### 2.5. Pre-Processing

**Figure 1:**
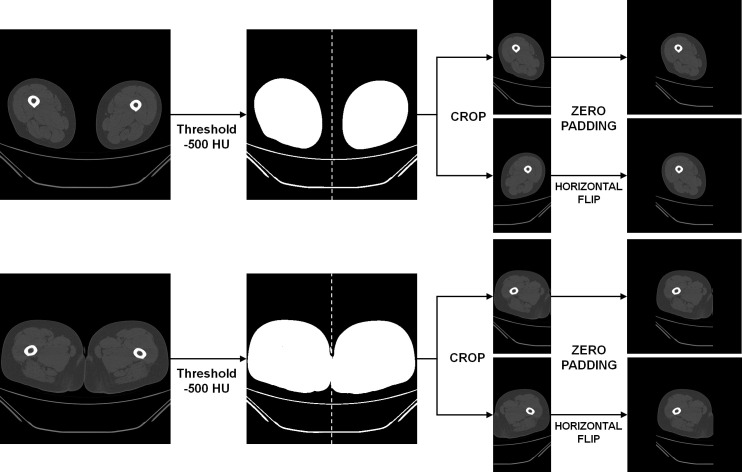
Pre-processing pipeline for thigh computed tomography (CT) images. Thresholding at −500 Hounsfield units (HU) was applied to create a binary mask separating air from other regions. The bilateral thighs were automatically identified, cropped, zero-padded to a uniform size, and horizontally flipped for data augmentation.

During the pre-processing stage, the left and right thigh regions were isolated from the CT scans, with each region subsequently utilized as independent data. In CT, it is recognized that air has an intensity lower than approximately −1000 HU, and fat tissue has an intensity ranging from −190 HU to −30 HU [21,22]. Pixels with attenuation values higher than −500 HU were extracted to generate a binary mask that separated air from other regions. Each connected region in the binary mask was automatically labeled, and its area was calculated. The component located at the uppermost position along the *y*-axis was designated as the reference region, and any component exhibiting an area smaller than half of the reference was deemed non-anatomical background (e.g., bed or sheets) and eliminated. Subsequently, the left and right thigh regions were cropped based on the center of the *x*-axis between the labels and separated into two images. The two separate images were zero-padded to the left and right, respectively, to ensure that the images possessed equivalent widths and heights [23] (Figure 1).

In the pre-processing stage, the left and right thigh regions were isolated and designated as distinct inputs. This approach was adopted to augment the number of available samples and to mirror the anatomical variability present at the regional level. Despite their shared origin, the thighs exhibit variability in muscle volume, fat distribution, and contour due to factors such as habitual loading, surgical history, or degenerative changes. This approach, therefore, provides more clinically realistic variation than conventional augmentation methods such as horizontal flipping, rotation, or scaling, which may introduce artificial symmetry or geometric distortion.

### 2.6. Ground Truth Labeling

Ground truth segmentation was performed on CT scans from all 136 patients in the internal dataset and 40 patients in the external validation cohort. The bilateral thighs of each patient were manually labeled by a trained radiologic technologist. Subsequently, an experienced musculoskeletal radiologist reviewed and corrected the labels to ensure accuracy and consistency.

The thigh muscles were divided into three functional groups: extensors, rectus femoris, vastus medialis, intermedius, and lateralis muscles; flexors, semimembranosus, semitendinosus, and biceps femoris muscles; and adductors, adductor longus, brevis, and magnus, sartorius, and gracilis muscles. The selection of these functional groups was driven by their representation of the primary muscle compartments responsible for knee extension, flexion, and adduction. These compartments hold clinical relevance for the evaluation of locomotor function and sarcopenia-related changes in the lower extremity [4,7]. The manual labeling of the three thigh muscle groups was performed on each axial image using in-house developed software implemented with Microsoft Visual Studio 2010 MFC (C++). The software facilitates slice-by-slice annotation and mask overlay visualization for quality control purposes, enabling precise delineation of each functional muscle group. The manual labeling process was conducted on a standard workstation that was equipped with a high-resolution medical-grade display, pixel-level zoom, and mask-overlay visualization. These tools enabled precise contouring for each slice. Manual segmentation was performed slice by slice by a trained radiologic technologist (Y.P.) and a radiologist (J.E.K., with two years of experience in musculoskeletal radiology). The final segmentation masks were exported in NIfTI (.nii) format, which is directly compatible with common medical image-processing and deep-learning frameworks such as PyTorch and MONAI. While the primary objective of this internal labeling tool is to ensure consistent file-naming conventions and labeling color codes across all cases, equivalent manual annotation could also be performed using open-source software such as 3D Slicer or ITK-SNAP, which produce compatible NIfTI outputs. Subsequently, a board-certified radiologist (J.E.K., with two years of experience in musculoskeletal radiology) reviewed all manually-segmented images and corrected the errors.

### 2.7. Training of the Deep Learning Model

In this study, a two-dimensional (2D)-based deep learning model was adopted. This decision was made due to the fact that the limited dataset size and computational resources were insufficient to train a full three-dimensional (3D) network. To this end, three distinct architectures (Dense U-Net, MANet, and SegFormer) were implemented and subsequently compared under identical training conditions. This approach was taken to evaluate the effects of convolution, attention, and transformer-based designs on thigh muscle segmentation performance.

The baseline model employed in this study was a Dense U-Net, a modified U-Net architecture that integrates DenseNet-style dense blocks into the encoder path. Each encoder block contained two convolutional layers (3 × 3 kernels) followed by batch normalization and ReLU activation, with 2 × 2 max-pooling for down-sampling. In the Dense U-Net, feature maps from all preceding layers within each encoder block were concatenated prior to the convolution operation. This facilitated efficient feature reuse and gradient propagation. The decoder blocks were configured to employ 2 × 2 transposed convolutions for up-sampling and concatenation with the corresponding encoder feature maps. This dense connection strategy has been shown to enhance the delineation of fine muscle boundaries and small anatomical details without substantially increasing model complexity [24,25,26,27]. The MANet architecture is an extension of the conventional U-Net framework. It incorporates multi-scale attention modules that adaptively weight spatial and channel features [28]. This mechanism enables the network to selectively emphasize relevant contextual information, thereby improving the segmentation of small or low-contrast muscle regions. The SegFormer model, a transformer-based encoder-decoder network, integrates a hierarchical vision transformer encoder with lightweight multi-layer perceptron (MLP) decoders [29]. This hybrid design facilitates the concurrent modeling of local details and global contextual dependencies, thereby ensuring robust generalization capabilities even with limited training data.

To ensure a fair comparison, all three networks were trained using the same training, validation, and external test datasets. The Adam optimizer and Jaccard loss function were utilized for optimization [30]. The batch size was set to four, considering GPU memory limitations (NVIDIA Tesla V100, 16 GB) and the high resolution of CT slices, a configuration commonly employed in medical segmentation tasks [25,27]. A three-step learning-rate decay was implemented, commencing at 1 × 10^−4^ for the initial phase, reduced to 1 × 10^−5^ during mid-training, and further decreased to 1 × 10^−6^ in the final phase to enable stable convergence and fine-tuning. The training was conducted for 250 epochs on an IBM Power System AC922 server that was equipped with four NVIDIA Tesla V100 GPUs (16 GB each) under Ubuntu 18.04.3.

In order to avert the occurrence of data leakage or optimistic bias, it was imperative that both thighs from a single subject were consistently allocated to the same subset, whether it was for training, validation, or testing. It was also crucial that the thighs were not divided across different subsets. An independent external validation cohort from another institution was additionally utilized to assess the generalizability of the models.

### 2.8. Volume Measurement

A trained deep learning model was utilized to segment the three muscle groups of the extensor, flexor, and adductor regions. The volume was measured by summing the number of pixels located within the region of each segmented muscle group. The volume was derived by multiplying the total number of pixels by the *x*-axis, *y*-axis pixel spacing, and slice thickness. Following the segmentation of the thigh muscle group, the tissues were classified based on HU. The use of HU scales facilitated the determination of body tissues through the quantification of muscle mass (HU = −29 to +150) and the subcutaneous, intermuscular, and intramuscular fat tissues (HU = −190 to −30) [31]. The total thigh volume, the volume of each muscle group, the volume of subcutaneous and intermuscular fat, the volume of intramuscular fat per muscle group, the lean muscle volume of the thigh, and the volume of each muscle group were obtained by multiplying the total number of designated pixels by the *x*-axis, *y*-axis pixel spacing, and slice thickness [32,33].

### 2.9. Statistical Analyses

The model’s performance was assessed by employing four statistical indices: sensitivity, specificity, accuracy, and DSC. The results of the deep-learning model were then compared with the ground truth data, and the true positive (TP), false positive (FP), true negative (TN), and false negative (FN) rates were calculated. The respective statistical indices were then determined.

A comparison was made between the volume as measured by the deep learning model and the volume of ground truth. This comparison was performed using Student’s *t*-test and Bland Altman plots, Pearson’s correlation, and Bland Altman plots. In addition, the reliability of the data was assessed through the implementation of an intraclass correlation coefficient (ICC) analysis. Statistically significant results were defined as two-sided *p* values less than 0.05. The analyses were performed using SPSS for Windows version 24.19.0 (SPSS Inc., Chicago, IL, USA).

## 3. Results

The automated segmentation of thigh muscles was performed using a deep learning-based approach, with the inference conducted on a GPU-equipped workstation (Intel Core i7-8700 @ 3.20 GHz, 32 GB RAM, NVIDIA GeForce GTX 1060 6 GB, Windows 10). The time range consumed per thigh volume from the ischial tuberosity to the patella bone level was between 75.9 and 123.83 s, with a mean time of 91.69 s. The results of the study encompassed not only the total muscle volume of the thigh but also additional values, including the lean muscle volume, the mean intramuscular fat fraction, and the extra-muscular fat volume. These additional values were automatically provided as results.

### 3.1. Internal Validation

**Figure 2:**
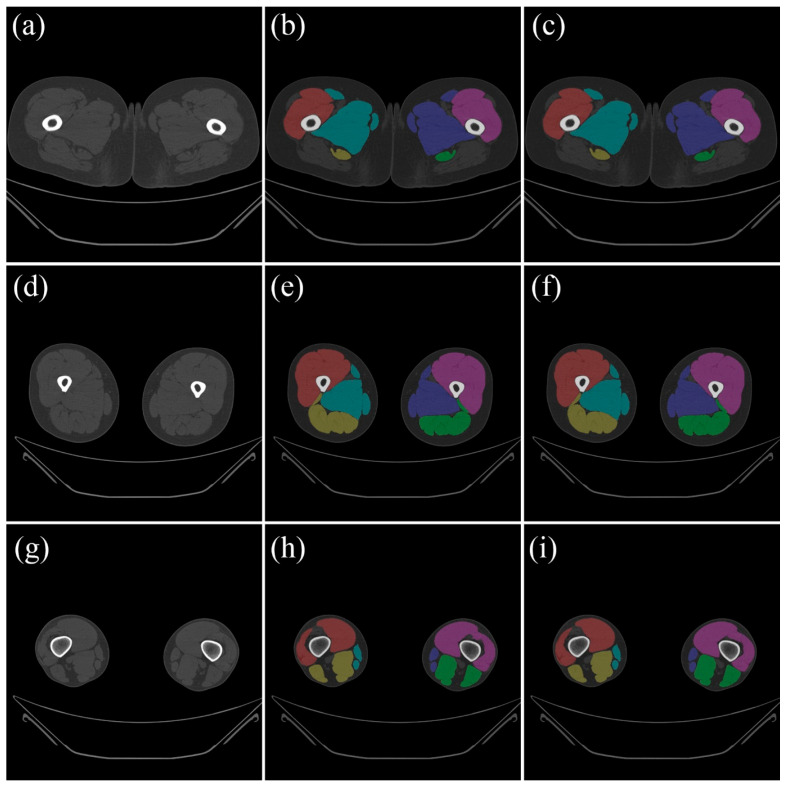
Examples of adductor, extensor, and flexor muscle segmentation results: (**a**,**d**,**g**) original images; (**b**,**e**,**h**) ground-truth annotations; and (**c**,**f**,**i**) deep learning-based automated segmentation results. In the segmentation results, adductor muscles are shown in red and *purple*, extensor muscles are shown in *teal* and *navy blue*, and flexor muscles are shown in *yellow* and *green*.

In this paper, internal validation was performed on test data that was constructed separately. Figure 2 compares the ground truth data from the test data with the results that were automatically segmented using the trained model.

To ensure the model’s robustness in terms of data dependency, we employed a five-fold cross-validation procedure [34]. The internal dataset comprised 42,274 CT slices from 136 patients, which were randomly divided into five equal subsets at the patient level [35,36]. In each iteration of the training process, four subsets, comprising approximately 33,692 slices, were utilized for training purposes, while a single subset, containing approximately 8582 slices, was allocated for the purpose of testing. The validation process was repeated five times, with each subset utilized precisely once as test data. The performance of this deep learning model was evaluated as the average of five validation results.

For the purpose of internal validation, the MANet achieved a mean DSC above 97% for all functional muscle groups (see Table 1). Specifically, the adductor, extensor, and flexor muscles exhibited mean sensitivities of 97.865 ± 2.532%, 98.706 ± 1.977%, and 97.584 ± 3.403%, respectively, along with mean DSCs of 97.732 ± 1.917%, 98.499 ± 1.656%, and 97.692 ± 2.607%, respectively. The three muscle groups exhibited specificity values greater than 99.9% and accuracies above 99.9%, thereby confirming precise boundary delineation with minimal false segmentation.

**Figure 3:**
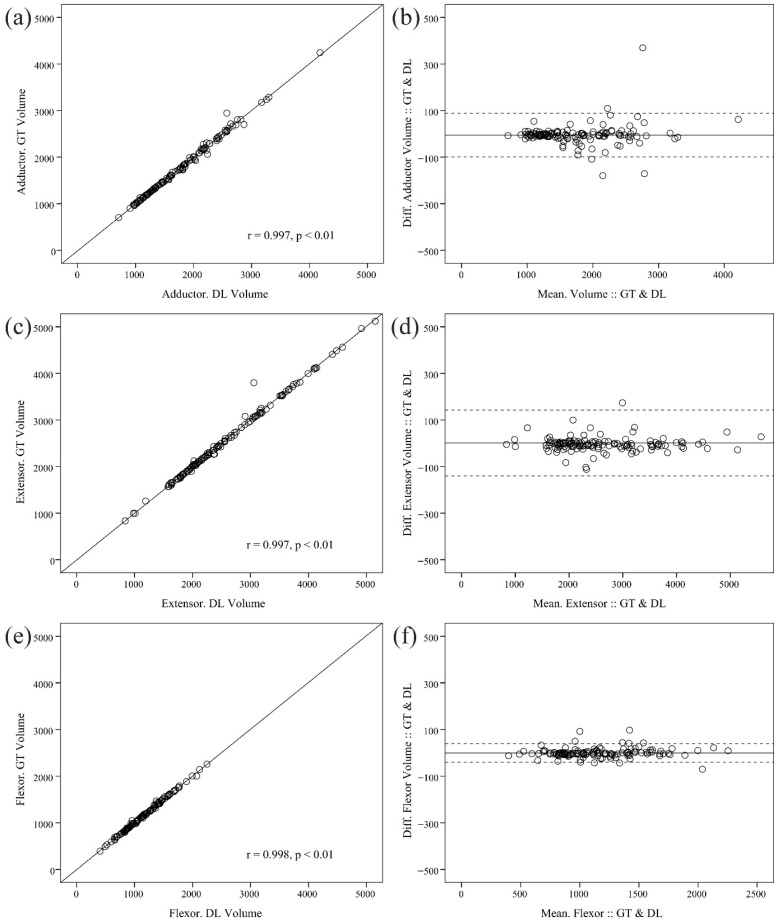
Scatter plots and Bland-Altman plots comparing ground-truth and deep learning-derived volumetric measurements on internal data: (**a**) scatter plot of adductor; (**b**) Bland-Altman plot of adductor; (**c**) scatter plot of extensor; (**d**) Bland-Altman plot of extensor; (**e**) scatter plot of flexor; and (**f**) Bland-Altman plot of flexor muscles.

The mean volumes determined by ground truth and deep learning showed no statistically significant differences (Table 2). All three architectures (Dense U-Net, MANet, and SegFormer) exhibited robust correlations between the predicted and manually measured muscle volumes. Among the models examined, the MANet model demonstrated the highest degree of agreement, with significant correlations between the ground truth and the volumes predicted by deep learning, as illustrated in Figure 3: (adductor: r = 0.997, *p* < 0.01; extensor: r = 0.997, *p* < 0.01; flexor: r = 0.998, *p* < 0.01).

Moreover, the ICC for the ground truth and deep-learning volumetric measurements in each muscle of the adductor, extensor, and flexor muscles indicates a high reliability of 0.999 (95% CI: 0.998~0.999, *p* < 0.01), 0.999 (95% CI: 0.998~0.999, *p* < 0.01), and 0.999 (95% CI: 0.999~0.999, *p* < 0.01), respectively.

Although Dense U-Net and SegFormer also achieved DSCs above 96%, the MANet slightly outperformed the others in overall accuracy and reliability, particularly for the extensor group.

### 3.2. External Validation

To assess the generalizability of the model, its external validation process involved the use of an independent dataset. This dataset comprised 4106 CT slices from 40 patients obtained from an external institution. The segmentation results and quantitative comparisons between manual and automatic measurements are summarized in Table 3 and Table 4. The MANet demonstrated mean DSCs of 97.474 ± 1.393%, 98.080 ± 0.841%, and 97.492 ± 1.347% for the adductor, extensor, and flexor muscles, respectively. The corresponding mean sensitivities were 98.035 ± 1.974%, 97.672 ± 1.469%, and 98.009 ± 1.885%, with specificity values consistently exceeding 99.9%.

**Figure 4:**
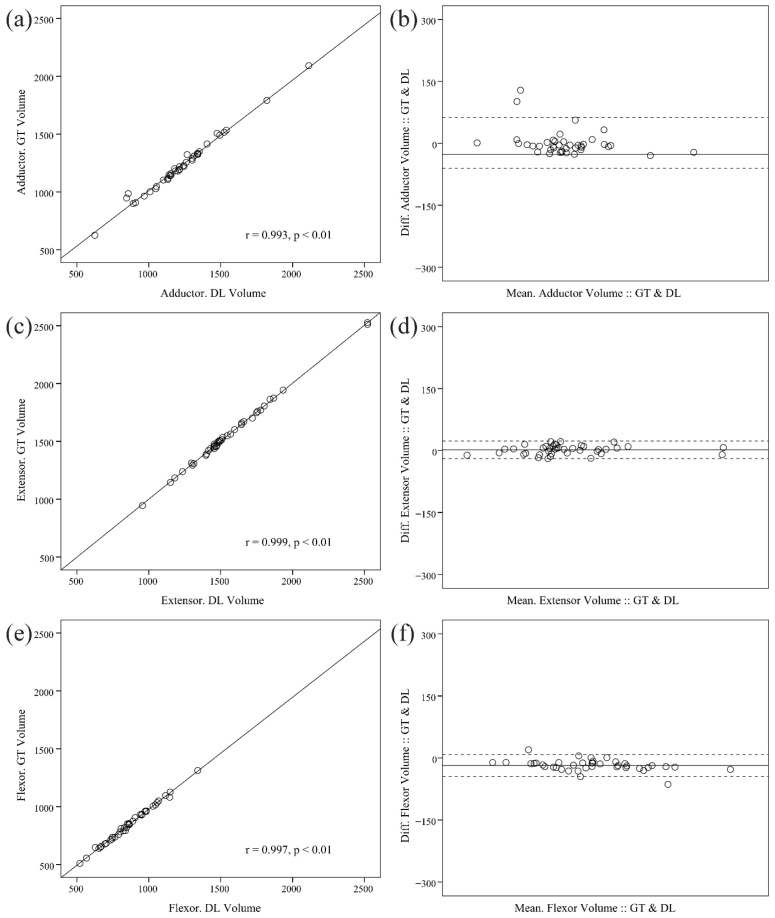
Scatter plots and Bland-Altman plots comparing ground-truth and deep learning-derived volumetric measurements on external data: (**a**) scatter plot of adductor; (**b**) Bland-Altman plot of adductor; (**c**) scatter plot of extensor; (**d**) Bland-Altman plot of extensor; (**e**) scatter plot of flexor; and (**f**) Bland-Altman plot of flexor muscles.

The mean muscle volumes as determined by the MANet and the ground truth were 1227.493 ± 261.478 cm^3^, 1561.149 ± 313.117 cm^3^, and 859.931 ± 176.221 cm^3^, respectively (Table 4). A one-way analysis of variance (ANOVA) was conducted to determine if there were statistically significant differences between the groups. The results indicated that there were no statistically significant differences (*p* > 0.05). All three architectures (Dense U-Net, MANet, and SegFormer) exhibited robust correlations between the predicted and manually measured muscle volumes in the external dataset. SegFormer demonstrated the highest volumetric agreement, while MANet and Dense U-Net exhibited comparable accuracy levels. The ensuing discussion will focus on the results of the MANet model, as presented in Figure 4. The model demonstrated a high degree of correlation for the adductor (r = 0.993, *p* < 0.01), extensor (r = 0.999, *p* < 0.01), and flexor (r = 0.997, *p* < 0.01).

Furthermore, the ICC for the ground truth and MANet-derived volumetric measurements in each muscle of the adductor, extensor, and flexor indicates a high reliability of 0.995 (95% CI: 0.993~0.998, *p* < 0.01), 0.999 (95% CI: 0.999~1.000, *p* < 0.01), and 0.998 (95% CI: 0.997~0.999, *p* < 0.01), respectively. Although SegFormer demonstrated marginally elevated ICC values for volume estimation, the MANet exhibited comparable performance and the highest mean DSC across all functional muscle groups. These findings suggest that both attention-based and transformer-based models possess the capacity to effectively generalize to external datasets, thereby substantiating the robustness of the proposed deep learning framework.

## 4. Discussion

A fully automated, deep learning-based algorithm was presented for the segmentation of thigh muscles into three distinct functional groups on non-contrast thigh CT images. This algorithm was developed to enable the quantification and qualification of thigh muscles with minimal computation time when executed on a GPU-equipped Windows workstation. The proposed deep learning models demonstrated comparable or superior segmentation accuracy compared to manual ground truth annotations, with an average Dice similarity coefficient of approximately 0.97. Among the three architectures evaluated (Dense U-Net, MANet, and SegFormer), the MANet exhibited the highest overall segmentation accuracy in internal validation, while the SegFormer demonstrated slightly superior volumetric agreement in external validation. These findings suggest that attention-based convolutional networks effectively delineate local anatomical structures, whereas transformer-based architectures provide improved generalization to heterogeneous datasets. The collective analysis of these results underscores the complementary characteristics of the evaluated architectures: convolutional attention-based networks, such as MANet, exhibit robust local feature discrimination, while transformer-based models, like SegFormer, demonstrate consistent performance across institutions. In future studies, a combination of local and global feature representations could enhance segmentation robustness and clinical applicability.

In this study, we demonstrated that deep-learning-based segmentation on CT can achieve a level of accuracy comparable to that of MRI-based reports from previous literature. The decision to employ CT rather than MRI was predicated on its extensive clinical availability, cost-effectiveness, and the feasibility of conducting opportunistic muscle-composition analysis from existing scans. A direct comparison between CT and MRI scans was not the objective of this retrospective study. However, it is a subject that will be explored in future studies that include cohorts of multimodal imaging. Although CT is not a standard diagnostic modality for musculoskeletal conditions due to concerns regarding radiation exposure (particularly in Europe), it is widely utilized for a variety of other clinical indications. Consequently, the strategic utilization of existing CT data facilitates quantitative muscle and fat analysis without the need for additional radiation exposure. Furthermore, advancements in deep learning-based image analysis, as exemplified by the approach delineated in this study, have the potential to expand the scope of CT’s application in musculoskeletal and metabolic research.

It is imperative to acknowledge that prior studies employing deep learning for muscle segmentation have been predominantly conducted on MRI. Conventionally, the segmentation of thigh muscles using deep learning has been predominantly conducted on MRI. This is primarily attributable to the enhanced soft tissue contrast provided by MRI in comparison to CT, thereby facilitating the segmentation process. However, CT has been demonstrated to offer a more cost-effective and versatile imaging modality than MRI. If CT can achieve a performance level comparable to MRI in segmenting thigh muscles, it could prove to be a valuable asset in clinical settings. A number of prior studies have endeavored to perform thigh muscle segmentation on CT scans. However, the utilization of thinner CT slices (1–2 mm) in comparison to MRI has presented challenges in the context of ground truth tasks, resulting in a constrained dataset and diminished performance. Therefore, there was a need to validate the feasibility of thigh segmentation with a larger number of CT scans. In the present study, we sought to undertake the segmentation of thigh muscles by leveraging a substantial dataset comprising 46,380 images from 172 patients. Consequently, we were able to achieve segmentation results that surpassed the performance of previous studies using MRI. This finding demonstrates that CT can be effectively utilized for thigh muscle segmentation, proving to be as viable an option as MRI.

It is imperative to acknowledge that the seemingly elevated Dice coefficients documented in our study must be contextualized within the framework of methodological variations across research endeavors. Conventional MRI-based studies have typically utilized smaller datasets, ranging from 10 to 110 cases, and frequently employed single-split validation or restricted cross-validation procedures. In contrast, our approach employs a substantially larger dataset, comprising 172 patients and 46,380 CT slices, and utilizes a five-fold cross-validation scheme. This approach ensures enhanced robustness and generalizability, thereby providing a more substantial foundation for reliable clinical decision-making. Furthermore, MRI provides superior soft-tissue contrast; however, it requires longer acquisition times and smaller cohorts. Conversely, CT, despite its lower contrast, allows standardized imaging and broader data availability across institutions. These factors likely account for the performance variation between modalities rather than true algorithmic superiority. Consequently, the findings of this study should be interpreted as demonstrating comparable or marginally enhanced performance in relation to MRI-based reports, rather than demonstrating direct superiority.

The primary impediment to the integration of CT body composition analysis into clinical practice is the absence of rapid and accurate, fully automated segmentation techniques. In recent decades, numerous fully automated techniques have been proposed, encompassing threshold-based approaches [37,38,39,40,41] and atlas- or knowledge-based approaches [42,43]. However, the majority of body composition analyses have been conducted in a single slice section, owing to the laborious nature of manual segmentation. Consequently, 3D volumetric analysis of body composition is more accurate and reliable than 2D approximation at one or a few levels. Furthermore, due to the intricate nature of the musculature of the thigh region and the comparatively lower soft tissue contrast offered by CT, the thigh muscles were typically extracted as a single entity rather than being segmented into their individual components. The present study proposes the integration of a fully automated segmentation system into clinical practice, with the objective of providing advanced anthropometric data on existing CT images. The implementation of this system is expected to facilitate the expeditious decision-making process within clinical settings. Furthermore, the offer of additional values is conceivable, including but not limited to lean muscle volume, mean intramuscular fat fraction, extra-muscular fat volume, and total muscle volume. It is hypothesized that the fully automated segmentation system will prove valuable in evaluating muscle quantity and quality in a variety of medical situations.

Recent studies have proposed the hierarchical multi-atlas method or the knowledge-based level set method for thigh muscle segmentation as an alternative to the time-consuming and challenging manual segmentation-based deep learning approach [41,42]. In instances where subjects possess a high degree of muscularity or in cases of particularly substantial muscle bellies, the delineation of boundaries between muscles becomes challenging, and the efficacy of the proposed methodologies in such scenarios is limited [42]. Moreover, manual segmentation of muscles is not feasible in such circumstances. In the present study, the thigh muscles were extracted into three functional groups: extensor, flexor, and adductor groups. This approach was taken instead of segmenting all muscles individually. The implementation of functional group segmentation could serve as a valuable strategy to circumvent the arbitrary segmentation that often occurs within the context of ambiguous intermuscular boundaries. Moreover, the proposed group segmentation approach has the potential to serve as an objective instrument for evaluating the clinical significance of each functional group of the thigh muscles in subsequent clinical studies, in comparison to isokinetic testing, which may be influenced by factors such as pain severity or the acute inflammatory phase.

The elevated level of segmentation accuracy attained by the proposed model is not only technically significant but also clinically meaningful. The objective assessment of sarcopenia, the monitoring of disease progression or rehabilitation outcomes, and the provision of reproducible biomarkers for treatment response in oncology or metabolic disorders can be facilitated by the automated quantification of muscle volume and fat composition from routine CT scans. Despite the absence of a direct evaluation of patient outcomes in the present study, the substantial agreement with manual segmentation lends credence to its potential utility in clinical decision-making and longitudinal follow-up.

The study’s limitations include the exclusive validation of the model on thigh CT scans. Although low-dose CT data were not included in this study, dose-dependent image noise could influence segmentation accuracy. A separate validation study is currently underway. This study utilizes low-dose protocols to assess the robustness and generalizability of the model. The objective of this assessment is to expand its clinical applicability and optimize radiation dose. It is imperative that we enhance our program to accommodate the variability in body muscle volumes, encompassing both CT and MR imaging modalities. Subsequently, the thighs were segmented into discrete images to augment the dataset. However, this approach is constrained by the relatively limited sample size of the patient population. In conclusion, although we provided body composition values such as lean muscle volume, mean intramuscular fat fraction, extra-muscular fat volume, and total muscle volume, we did not have any objective references for them. Further research is necessary to compare the CT-derived values with those obtained from DXA or MRI. Furthermore, the retrospective dataset did not contain BMI information, which constrained the analysis of segmentation performance according to body habitus. Subsequent prospective studies that incorporate anthropometric indices, such as BMI, will assist in elucidating how variations in body composition may impact the precision and generalizability of CT-based muscle segmentation.

Beyond the technical performance, the proposed model demonstrates substantial potential for clinical translation. The extraction of muscle and fat metrics from routine CT scans via automated processes holds potential for opportunistic assessment of sarcopenia, frailty, and treatment response without incurring additional radiation exposure or financial costs. The integration of this framework into hospital PACS or AI platforms has the potential to facilitate real-time quantification of muscle composition, thereby aiding in the development of personalized rehabilitation plans and prognostic evaluations. From a research standpoint, expanding this approach to whole-body analysis and multimodal imaging (including MRI) may facilitate the establishment of generalized foundation models for muscle segmentation and body-composition assessment across institutions and populations. These advances have the potential to facilitate precision health strategies and broaden the clinical utility of musculoskeletal imaging. A comprehensive review of the extant literature reveals a preponderance of evidence that lends support to the hypothesis that CT-based deep learning models possess a high degree of feasibility for the purposes of practical and scalable muscle assessment in both clinical and research settings.

Although inter-observer variability was not quantitatively analyzed, all manual segmentations were first created by a trained technologist and subsequently reviewed and corrected by an experienced musculoskeletal radiologist to ensure consistency. The proposed model demonstrated an ICC greater than 0.99 in comparison with the manual segmentation approach. This result is analogous to, or exceeds, the inter-observer agreement levels reported in extant muscle segmentation studies. Subsequent studies will incorporate multi-observer validation to provide additional confirmation of clinical reliability.

## 5. Conclusions

In conclusion, a fully automated deep learning-based muscle segmentation of the thigh into three extensor, flexor, and adductor groups was successfully achieved, very similar to manual segmentation. This method could be easily used to evaluate thigh muscle quantity and quality for each functional group of the thigh.

## Figures and Tables

**Table 1:** Results of internal validation for the deep learning-based thigh muscle segmentation method.

		Sensitivity (%)	Specificity (%)	Accuracy (%)	DSC (%)
Adductor	U-Net	97.741 ± 2.179	99.958 ± 0.034	99.923 ± 0.064	97.551 ± 1.899
	MANet	97.865 ± 2.532	99.973 ± 0.037	99.950 ± 0.050	97.732 ± 1.917
	SegFormer	96.696 ± 2.883	99.964 ± 0.029	99.936 ± 0.041	96.590 ± 2.174
Extensor	U-Net	98.187 ± 2.364	99.962 ± 0.025	99.924 ± 0.086	98.156 ± 1.773
	MANet	98.706 ± 1.977	99.972 ± 0.025	99.951 ± 0.032	98.499 ± 1.656
	SegFormer	98.362 ± 1.783	99.963 ± 0.022	99.934 ± 0.029	98.216 ± 1.502
Flexor	U-Net	97.693 ± 2.168	99.977 ± 0.016	99.954 ± 0.037	97.631 ± 1.837
	MANet	97.584 ± 3.403	99.987 ± 0.015	99.971 ± 0.027	97.692 ± 2.607
	SegFormer	97.469 ± 2.448	99.978 ± 0.017	99.956 ± 0.028	97.438 ± 1.874

U-Net, u-shaped convolutional encoder-decoder network; MANet, multi-scale attention network; SegFormer, segmentation transformer; DSC, dice similarity coefficient.

**Table 2:** Comparative analysis between ground-truth and deep learning-derived volumetric measurement results on internal data.

		GT Mean Volume	DL Mean Volume	*p* Value *	ICC	*p* Value **
Adductor	U-Net	1759.538 ± 611.622	1764.705 ± 607.037	0.945	0.998	<0.01
	MANet		1762.371 ± 610.118	0.976	0.999	<0.01
	SegFormer		1763.093 ± 611.532	0.957	0.998	<0.01
Extensor	U-Net	2588.692 ± 882.738	2587.574 ± 876.641	0.992	0.998	<0.01
	MANet		2588.010 ± 885.939	0.997	0.999	<0.01
	SegFormer		2588.574 ± 879.111	0.996	0.999	<0.01
Flexor	U-Net	1141.467 ± 352.777	1141.939 ± 351.005	0.991	0.999	<0.01
	MANet		1141.822 ± 353.043	0.993	0.999	<0.01
	SegFormer		1141.901 ± 350.995	0.990	0.999	<0.01

U-Net, u-shaped convolutional encoder-decoder network; MANet, multi-scale attention network; SegFormer, segmentation transformer; GT, ground truth; DL, deep learning; ICC, intraclass correlation coefficient. * *p* value for Student’s *t*-test, ** *p* value for ICC.

**Table 3:** Results of external validation for the deep learning-based thigh muscle segmentation method.

		Sensitivity (%)	Specificity (%)	Accuracy (%)	DSC (%)
Adductor	U-Net	97.238 ± 2.889	99.968 ± 0.009	99.935 ± 0.033	97.309 ± 1.603
	MANet	98.035 ± 1.974	99.963 ± 0.027	99.941 ± 0.033	97.474 ± 1.393
	SegFormer	98.153 ± 1.086	99.941 ± 0.026	99.910 ± 0.027	97.537 ± 0.723
Extensor	U-Net	97.950 ± 0.533	99.970 ± 0.008	99.938 ± 0.013	97.998 ± 0.481
	MANet	97.672 ± 1.469	99.976 ± 0.021	99.935 ± 0.023	98.080 ± 0.841
	SegFormer	97.090 ± 0.958	99.979 ± 0.012	99.921 ± 0.020	98.002 ± 0.472
Flexor	U-Net	98.445 ± 1.648	99.968 ± 0.012	99.956 ± 0.018	97.412 ± 1.321
	MANet	98.009 ± 1.885	99.978 ± 0.016	99.961 ± 0.024	97.492 ± 1.347
	SegFormer	97.106 ± 1.453	99.978 ± 0.015	99.954 ± 0.025	97.141 ± 0.830

U-Net, u-shaped convolutional encoder-decoder network; MANet, multi-scale attention network; SegFormer, segmentation transformer; DSC, dice similarity coefficient.

**Table 4:** Comparative analysis between ground-truth and deep learning-derived volumetric measurement results on external data.

		GT Mean Volume	DL Mean Volume	*p* Value *	ICC	*p* Value **
Adductor	U-Net	1226.797 ± 251.731	1226.102 ± 261.369	0.990	0.996	<0.01
	MANet		1227.493 ± 261.478	0.990	0.995	<0.01
	SegFormer		1226.899 ± 251.578	0.990	0.996	<0.01
Extensor	U-Net	1563.165 ± 303.122	1561.297 ± 301.901	0.978	1.000	<0.01
	MANet		1561.149 ± 313.117	0.971	0.999	<0.01
	SegFormer		1562.346 ± 294.874	0.989	1.000	<0.01
Flexor	U-Net	847.986 ± 167.578	866.123 ± 172.698	0.635	0.998	<0.01
	MANet		859.931 ± 176.221	0.752	0.998	<0.01
	SegFormer		857.527 ± 172.671	0.801	0.999	<0.01

U-Net, u-shaped convolutional encoder-decoder network; MANet, multi-scale attention network; SegFormer, segmentation transformer; GT, ground truth; DL, deep learning; ICC, intraclass correlation coefficient. * *p* value for Student’s *t*-test, ** *p* value for ICC.

## Data Availability

The restriction is due to patient privacy protection and institutional review board (IRB) regulations. The imaging data used in this study contain sensitive clinical information, and redistribution is not permitted under our IRB approval. Therefore, the dataset can only be shared by the corresponding author upon reasonable request for research purposes, after appropriate review and approval.
